# Traditional Acupuncture Meets Modern Nanotechnology: Opportunities and Perspectives

**DOI:** 10.1155/2019/2146167

**Published:** 2019-07-16

**Authors:** He Zhang, Gang Han, Gerhard Litscher

**Affiliations:** ^1^Department of Respiration, Guang'anmen Hospital, China Academy of Chinese Medical Sciences, Beijing 100053, China; ^2^TCM Research Center Graz, Research Unit of Biomedical Engineering in Anesthesia and Intensive Care Medicine and Research Unit for Complementary and Integrative Laser Medicine, Medical University of Graz, 8036 Graz, Austria; ^3^Department of Biochemistry and Molecular Pharmacology, University of Massachusetts Medical School, Worcester, MA 01605, USA

## Abstract

Acupuncture is an ancient method in traditional Chinese medicine (TCM). Usually acupuncture needles are inserted into the body to achieve therapeutic effects. However, there are still some challenges to achieve consensuses. What is the essence or anatomy of acupuncture meridians? How does acupuncture work? How to improve acupuncture clinical therapeutic effect? These questions may be addressed by highlighting recent developments in innovative nanotechnology. The aim of this review is to elucidate the possible applications and future potential of nanotechnology in acupuncture. Nanoparticles are promising for imaging and it may gain a better understanding of the essence of meridian. Nanotechnology enables nanochips/nanosensors providing new solutions in detection reactive molecules* in vivo *and in real time. The connections and changing of these molecules with needle stimulation will allow insight into the mechanisms of acupuncture. Acupuncture combined with nano-TCM could provide a great potential in some type of characteristic acupuncture therapies improvement. By virtue of nanotechnology, the acupuncture needles could be innovated as multifunction toolbox. Acupuncture needles could be considered as a method for controlled drug delivery. The nanoparticulated photothermal, magnetothermal, photodynamic agents could also be filled on the surface of needle.

## 1. Introduction

Acupuncture is an ancient typical therapy in traditional Chinese medicine (TCM), in which acupuncture needles are inserted into the body to achieve desired therapeutic effects [1]. Acupuncture is believed to originate in China. Thousands of years have witnessed the remarkable clinical applications of acupuncture. According to the World Health Organization (WHO), acupuncture has been shown to be an effective alternative or complementary treatment to 28 diseases, symptoms, or conditions [2]. In daily practices, the clinical applications of acupuncture spread far beyond these diseases. Moreover, acupuncture is suitable for the large majority, regardless of physical condition, with little or no side effect. Despite the long history and widespread use of acupuncture, there are still some challenges in achieving a consensus. The efficacy of acupuncture remains a topic of discussion. Some studies, including multicenter randomized control trials (RCT), have suggested that acupuncture is no more than a theatrical placebo [3–5]. Besides, how acupuncture works as well as the molecular mechanisms behind it is still unclear. The meridian theory has a solid role in guiding the clinical practice of acupuncture and is a fundamental of TCM science. However, as of now, the anatomy of meridians has not been explicated in detail [6]. Acupuncture also has the feature of TCM treatment based on syndrome differentiation. By selecting different acupoints and changing the manipulation of the system, a unique, but different, clinical outcome results each time. Therefore, it is becoming increasingly necessary to standardize acupuncture treatment [7]. These challenges are consequently the key roadblocks hindering the advancement of acupuncture. Thus, new and modern technologies are essential to draw the mysterious veil and promote the development of traditional acupuncture [8].

**Figure 1:**
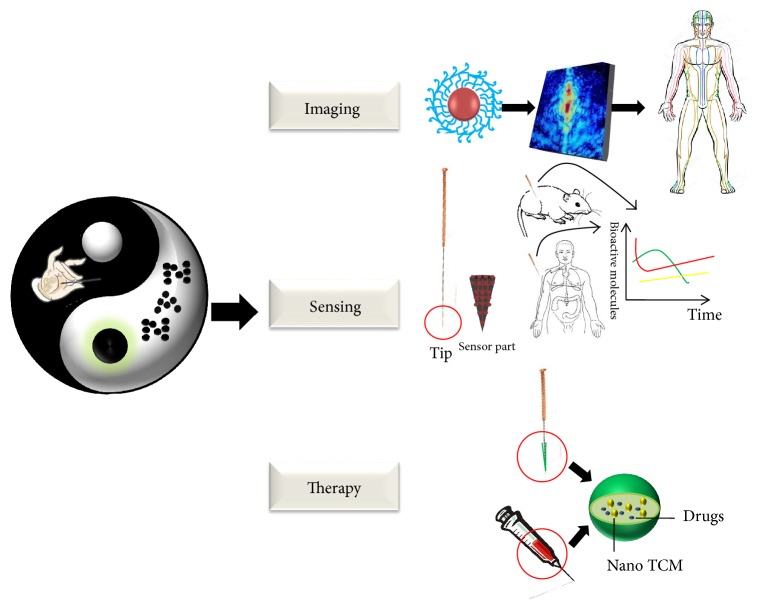
Schematic diagram of some applications and potential of nanotechnology in acupuncture.

Nanotechnology is the engineering of materials that have at least one dimension sized from 1 to 100 nm for technological or scientific applications [9]. In 1959, Richard Feynman, considered the father of nanotechnology, presented the famous lecture entitled “There's Plenty of Room at the Bottom” and introduced the idea of manipulating atoms and molecules by using nanoscale machines [10]. The term “nanotechnology” was first used by Norio Taniguchi in 1974 [11]. Since then, this emerging field has evolved dramatically and is now being applied to various fields including nanomedicine, nanoelectronics, biomaterials, energy production, and information technology. The brilliance of nanotechnology lies in the size of the nanoparticles. Reducing the size of the structure to the nanoscale results in completely different properties, like unique performances in fields such as chemistry, photology, electricity, and magnetism [12–14]. Due to these attractive properties, nanoparticles have been widely used for biological applications such as biomarkers, imaging, and biosensors. These applications have laid an important foundation to reveal the underlying mechanism of acupuncture and innovate the acupuncture needles. This review will attempt to highlight the possible applications and potential of nanotechnology in acupuncture, as shown in Figure 1.

## 2. Seeking the Essence of Meridians

The meridians should be pathways along which the life energy, known as* Qi*, is transported [15].* Huangdi Neijing* (Yellow Emperor's Classic of Internal Medicine) provides a systematic illustration of the theory and methods of acupuncture and moxibustion. Meridian system is an important component of the theoretical system of TCM, consisting of the main meridian channels (Jing Mai) and the collateral vessels (Luo Mai). Jing Mai, main branches of the system, contain twelve main meridians, eight extra meridians, twelve divergent channels, and twelve sinew channels, as well as twelve cutaneous regions. The Luo Mai, a branching expanse of capillary-like vessels which spread throughout the body, comprises fifteen large collaterals that connect the twelve main meridians in various ways, superficial collaterals, and tiny collaterals. The comprehensive, complex meridian system should supply* Qi* to every part of the body. Acupoints, lying along the meridians, can be the sites from which the* Qi* of organs and meridian is transported to the body surface. Meridian theory has been applied for guiding acupuncture clinical practice for thousands of years. Hence, defining meridians essence is a key scientific issue. In fact, some phenomena of skin diseases and migration of isotopes along the meridians have confirmed some notes for the existence of the meridians to some extent [16, 17]. Researchers never stop seeking the material basis of acupuncture. Some results demonstrated that the physical character of the meridians mainly included low resistance [18], high conductivity [19], the phenomenon of temperature distribution [20], high luminosity [21], and other physical phenomena [22, 23].

However, in the near past, the most sensational research on anatomy of acupuncture meridians went back to the early 1960s. Kim Bonghan, a North Korean professor, declared that their research team found the anatomic and physiological basis of the meridians [24]. A new system was proposed, constituting nodes like anatomical structures at the acupoints and the tube-like structure connected to the nodes in the skin. The nodes and the tubes were named Bonghan corpuscles and the Bonghan ducts. Bonghan liquor was discovered, a liquid circulating in the Bonghan ducts, which is abundant in DNA, RNA, hyaluronic acid, more than 19 amino acids, and 16 free mono nucleotides [24]. Besides, according to Kim's reports, the Bonghan system comprised various biochemicals related to immune activities and “Sanals” with self-regenerating properties [25]. According to Sanal theory, the amount of DNA contained in one Sanal is nearly equal to that contained in one chromosome [26]. Kim's results created a great sensation at that time. However, unfortunately there were several attempts to reproduce Kim's results but without success [26]. Thus, Kim was thought to cheat in his research and apparently he did not describe the materials and methods in detail. Around 40 years later, the story dramatically reversed and Kim's discoveries were confirmed by a Korean researcher Kwang-sup Soh. Kim's team holds the views that the threadlike microscopic anatomical structure as primo vascular system (PVS) is served as an extension of acupuncture meridians [25]. Bonghan duct, corpuscle, and Sanals were renamed: primo vessel (PV), primo node (PN), and primo-microcells, respectively [24, 27].

The generation of diverse type nanoparticles clearly exhibited the importance of nanoparticles in biological imaging applications [8, 28, 29]. Apart from Trypan blue spraying technique, fluorescent nanoparticles, gold nanospheres, and quantum dots were adopted to visualize the novel system. Hollow gold nanospheres (HGNs) can be adjusted to generate an optical contrast to the intralymphatic PVS (IL-PVS) because of the porous nature of the PV's external wall [30]. As previously stated, the diameter of the pore was smaller (~1 *μ*m) than the external diameter (2–5 *μ*m) in rabbits, suggesting that lymph inflow into PVs was relatively easier than outflow [31]. Eric Carlson et al. [30] selected two ranges of the HGNs size, which were 50–70 nm and 100–125 nm. The HGNs colors were turquoise and green for the 50–70 nm and 100– 125 nm, respectively. The two different sizes of HGNs were injected into lumar lymphatic nodes (LLN). When the HGN-contrasted PV became visible, it was still need to further confirm it as an IL-PV. The result showed both the LLN and LV darkened after HGNs injection, but LVs remain partially translucent allowing visualization of IL-PVS. The turquoise-green-colored HGNs seemed to provide excellent optical contrast for the IL-PVS in rats. Kwang-sup Soh holds the view that the structure of PVS was very similar with the connective tissues around the PVS so that it could not be discriminated in the conventional histology. However, the fluorescence nanoparticles were preferentially absorbed by the primo vessels compared with surrounding tissues. In order to elucidate the functional and the medical aspects of the PVS, fluorescent nanoparticles could be used as contrast agents. Several kinds of fluorescence nanoparticles have been developed by using silica loading fluorescence dyes, including the rhodamine B isothiocyanate [32–34], and a mixed dye of commercial Pelikan ink and rhodamine B [35] to observe the PVS in the abdominal wall, epineurium along the sciatic nerve, the subcutaneous layer of skin, and even the fourth ventricle and spinal cord. These nanoparticles were detectable with MRI [36]. In order to evaluate the feasibility of using nanoparticles as a contrast agent during MRI or CT imaging of PV in the brain or the spinal cord, the nanoparticles were injected into the lateral ventricles of rats. The nanoparticles, flowing from lateral ventricles to the fourth ventricle and the central canal of the spinal cord, were absorbed by the primo vessel floating in the cerebrospinal fluid (CSF). The florescence of the nanoparticles was measured and calibrated by using a reference suspension. The results showed that the nanoparticles could be potential as a contrast agent to observe PV in the brain or the spinal cord. The magnetic fluorescent nanoparticles with proper surface modifications are not only nontoxic and biocompatible but also targetable to the specific area under external magnetic field [37, 38]. Johng et al. observed that magnetic fluorescent nanoparticle flew along the liver meridian, where the route was same as the classical books recorded, from the acupoint Taichong (LR 3) to the acupoint Yinbao (LR 9) where a magnet was attached to the skin surface [39]. In addition, IL-PVS, a novel threadlike structure inside the lymphatic vessel, could be imaged* in vivo *by the magnetic fluorescent nanoparticle [40, 41]. The nanoparticles were injected into two lumbar lymph nodes and a magnet was placed on the lymphatic vessels which were connected to the nodes. However, injection into lymph node to visualize PV and PN is not always recommended as bleeding is not avoidable. The best method to verify the existence of PV and PN is to directly inject some dyes or nanoparticles into lymphatic vessels, not lymph node of the rabbit. In the case of rat, actually, the size of the lymph vessel is not enough to be controlled* in situ* and* in vivo* for visualization. It further has to be mentioned that nanoparticles could be spread into fibrin fibers which frequently are formed inside lymph vessel owing to even slight bleeding.

In terms of the external magnetic field, the injected nanoparticles were attracted and stayed inside the lymphatic vessel rather than flowing away with the lymphocytes. Thus, nanoparticles were taken up by the threadlike structures. According to Soh's research, we can surely gain a revelation that there are still some novel structures which are not noticed or recognized in numerous conventional histological studies. Soh's team believed that PVS is an extension of acupuncture meridians. The PVS is a previously unknown system indeed, but the question what the relationship between PVS and meridians is still needs future exploration. To a certain extent, the difference of distribution, position features, identification, and origin of PVS are still inconsistent with classic acupuncture meridian theory [42].

Within this article, the PVS are discussed as example and to highlight the possible applications and potential of nanotechnology in acupuncture. Maybe the PVS is lack of mainstream acceptance, but the research methods could provide reference. In this review, the PVS are described according to the published articles without overstating it. We clearly want to point out that the relationship between PVS and meridians still needs future exploration. This is in accordance with the article of Kang et al. [43]. These authors also reported on identifying PVS with nanoparticles; however they also stated that the possible existence of PVS is still not known to many scientists [43].

## 3. Detecting the Biological Mechanisms of Acupuncture

Another debate of acupuncture in the medical community is how the acupuncture works and what its underlying molecular mechanisms are. Based on TCM theory, disease is caused by deficiencies, excess, or imbalances of* Qi*. Thus, stimulating the specific acupoints could regulate corresponding organs or meridians and subsequently restore the state of balance. Much modern scientific work has been done to seek the mechanism of acupuncture. Unfortunately, there is no unified theory of acupuncture mechanism. Various models and hypotheses for different clinical applications have been proposed. In summary, mechanisms of acupuncture treatment are included in neurobiological mechanism by which acupuncture may trigger a somatic autonomic reflex, changing the levels of neurotransmitters, affecting the hypothalamus pituitary axis [44–46] and immunomodulatory effects [47], and modulating neuro-endocrine-immune (NEI) network [48].

Acupuncture needles are vital tools in the delivery of acupuncture, coming in different diameters and lengths to be used on the different areas of the body. The earliest acupuncture needles were named* bian* stones made of sharp edged stones. In ancient times, people lived in a chilly outdoor environment. The heated sharp stones were found as instruments of healing by scratching or pricking the certain site of body [49]. Since then, the theories of acupuncture, such as description of the meridians, acupoints location, and needling techniques, have emerged gradually. The materials of acupuncture needles also experienced great development during thousands of years.

Early acupuncture needles were made from the bamboo and bone. With the emergence of metallurgy, iron, copper, bronze, silver, and gold needles were made. However, compared with other materials, stainless steel needles are more economical and practical in clinical practice. Due to the property of good conductivity, acupuncture needles can be considered as a modern biosensor or other innovative tools.

Nanotechnology enables nanosensors providing new solutions in physical, chemical, and biological sensing and largely increases detection sensitivity, specificity, and portability. It laid a foundation for detection reactive molecules* in vivo* and in real time. Furthermore, it supports an excellent tool for observing and studying the biological effect and biological function of these reactive molecules. The connections of these molecules with the needle stimulation at the central and peripheral level will absolutely allow insight over the mechanisms of acupuncture treatment. The use of carbon nanotubes (CNTs) for sensing applications is promising [50]. It has already been reported that CNTs are used to measure and acquire pH, temperature, and biomedical information [51–53]. In order to monitor the molecular event occurring in acupoint and gain access to the local responses of acupuncture stimulation, a unique nanosensing platform by modifying carbon nanotubes (CNTs) on the tip surface of acupuncture needle has been developed. The CNT-modified acupuncture needle (CNT/AN) monitored serotonin (5-HT) level* in vivo *via electrochemistry in acupoint* Zusanli *(ST 36) [54]. The 5-HT was pumped into the acupoint ST 36 to make a proof-of-concept experiment. The results indicated that the CNT/AN was quite stable and able to detect the neurotransmitters* in vivo*, showing a great potential for better understanding the mechanism of acupuncture treatment. However, in fact, the effects of acupuncture on the healing process are through a holistic pathway, producing a series of physiological reactions, not only depending on one simple bioactive molecule or one signaling pathway changing. Thanks to the nanotechnology and Omics-based techniques, it may give answers to the acupuncture complex mechanisms. Omics-based techniques refer to screen of targets from nucleic acids to proteins and metabolites and their heterogeneous interactions. As the development of the technology of nanobiochips and omics, it will be possible to integrate relatively high spatial (different body regions and tissues) and temporal resolution data to reveal the molecular signaling pathways that flow from the tip of the needle to the disease or injury site in the near future [55]. The information obtained from acupoint, the target organ, as well as systemically, can be enriched with the temporal resolution data to construct a systems biology network. This network will provide a holistic view of how acupuncture works and its potential mechanism. Raman spectroscopy is commonly used in chemistry to provide a structure fingerprint based on vibrations in molecules [56]. It is also considered as a qualitatively or semiquantitatively analytical tool with the function of ultrasensitive detection because of the design and fabrication of nanostructure with surface-enhanced Raman scattering (SERS) activity. With the merits of being minimally invasive [57], the acupuncture needle was used as a tool, coated on the gold nanoshells (GNSs) and polystyrene, which could absorb SERS-active nanomaterials to become a SERS-active needle [58]. When inserted into the body, the SERS-active needle can detect interstitial fluids. And when SERS-active needle was pulled out, analytes in the diffused fluids at different depths were taken out in the meantime. The SERS-active needle presented an approach on depth profiles of target molecules in tissues. A novel SERS-active needle has the function of detection glucose* in vivo *by integrating glucose oxidase (GOx, signal convertor), 4-mercaptobenzoic acid (4-MBA, signal reporter), and microporous polystyrene [59]. This method could be a universal strategy to SERS detection of small biomolecules* in vivo *with the suitable integrating enzymes and corresponding reporters on SERS-active needles.

An important aspect of acupuncture treatment is that acupuncture needle must be manually manipulated to get the* Deqi *response after insertion into the body.* Deqi *is considered as an important component in the process of achieving therapeutic effectiveness in the acupuncture treatment. The* Deqi *feeling of patients is individual difference, which can be described as* suan *(aching or soreness),* ma *(numbness or tingling),* zhang *(fullness or pressure), or* zhong *(heaviness) around the acupuncture point or along the meridians [60]. When* Deqi *occurs, the practitioner, meanwhile, perceives a sensation often called “needle grasp.” However, the underlying mechanisms of acupuncture manipulation and the biological effect of needle grasp have remained unresolved. The hypothesis of the needle grasp mechanism might involve the winding connective tissue during the process of needle manual manipulation, which might deliver a stronger mechanical signal into the tissue [61]. A research has been conducted that used silicon carbide sandpapers with different grit numbers to manipulate the needle grasp force by changing surface roughness. The atomic force microscope (AFM) images were obtained by using Nanostation II™. AFM, as one of the foremost imaging tools, can measure and manipulate matters at the nanoscale by using a high-resolution scanning probe. In order to observe and verify the scratches on the surface of the needle analytically, the shape and depth of the scratch were analyzed. The result showed that surface roughness of the acupuncture needle could enhance the analgesic effect of acupuncture therapy, which partially supports the mechanical signaling theory through the winding connective tissues in the process of acupuncture manipulation [62]. To get the* Deqi *response, acupuncture needles are manually manipulated after insertion into the body. The typical acupuncture manipulation is made up by rapid twisting (back and forth or opposite direction) and lifting-thrusting (up and down motion) of the needle [63]. The stimulation amount and intensity are significant factors for different amount and intensity of simulation cause different physiological effects. However, these acupuncture manipulations are merely based on the patient's feelings and there is a lack of a quantitative, objective standard. Some efforts have been made to attempt to solve the problem. The force sensor (Nano-17 titanium) was attached to the acupuncture needle, and various rotation frequencies or lifting-thrusting movements were measured after acupuncture needle inserting in phantom tissue [64, 65]. While maybe it would be better to use the nanosensor which was connected to the tip of acupuncture needle; in that case, the real force and the amount of stimulation inside the tissue could be measured. Or even the acupuncture needle tip could be connected to multisensors, by which the force, the amount of stimulation, and therapy-induced biochemical progression could be monitored in real time and* in vivo*.

## 4. Enhancing the Therapeutic Effect

Acupuncture, as one of the complementary and alternative therapies, has attracted much attention to its therapeutic effect. How to enhance the clinical therapeutic effect and meanwhile reduce the discomfort induced by acupuncture needles are challenging and considerable. Clinically, various needle parameters such as diameter, depth of insertion, and number of needles used at a time are important factors for improving acupuncture performance. Some studies suggested that applying thick needles or deeper insertion could increase stimulus intensity and ultimately achieve the desired level of* Deqi *[66]. Thus, based on these parameters, a novel class of acupuncture needles, porous acupuncture needles (PANs) with hierarchical micro-/nanoscale conical pores upon the surface, have been fabricated [67]. The surface area of PANs was approximately 20 times greater than conventional acupuncture needles. The conventional acupuncture needles and PANs were inserted into the receptive field of the lumbar spinal dorsal horn neuron in alcohol dependence Wistar rats. The neuronal response to acupuncture stimulation, tremor activity, and Cocaine induced locomotor activity were investigated. Furthermore, the comparison of the different needles was made via measurement of spontaneous pain sensation induced by acupuncture needle insertion. The results demonstrated the higher efficacy of PANs in psychiatric treatment over conventional needles. Further, the PANs showed reduced pain sensation levels compared with the same interfacial surface area of conventional acupuncture needles. The fabrication of these novel PANs could be good news for the people fearing pain.

Acupoint injection therapy is an innovation in TCM where herbal extract or liquid drug was injected into acupoint for treatment or prevention [68]. Bee venom (BV), consisting of various biologically active compounds, is one of the most commonly encountered animal venoms [69]. BV injection exerts a remarkable effect in chronic diseases such as shoulder pain and inflammation, with the function of stimulating an acupoint as well as inducing allergic reactions in the human body [70–72]. Although the therapeutic utility of BV injection has been demonstrated, its safety profile is still somewhat limited. A systemic or local allergic response, accompanying fever, tonic pain, and edema, could occur after the BV injection [73]. To enhance the target or delivery efficiency of the drug, thus reducing the side effect of BV injection, the nanoparticle-based drug delivery systems seem to be a promising strategy. Jeong et al. [74] investigated the BV loaded into biodegradable poly(d,l-lactide-co-glycolide) nanoparticles (BV-PLGA-NPs) on formalin-induced pain. BV-PLGA-NPs were injected into the acupoint Zusanli (ST 36) at 0.5, 1, 2, 6, 12, 24, and 48 h before plantar injection of formalin. BV-PLGA-NPs exhibited the same analgesic effect as typical BV injection at the time points of 0.5, 1, and 2 h but provided a more prolonged effect than typical BV acupuncture treatment. Compared with classical acupuncture, acupoint injection therapy is easily administered, standardized, and timesaving [75]. However, at the moment the type of drugs for acupoints injection such as BV, placental extract, and Chinese medicine injections are limited [76]. Thanks to nanotechnology, a new concept, nano-traditional Chinese medicine (nano-TCM) has been proposed. Nano-TCM refers to bioactive ingredients, bioactive parts, medicinal materials, or complex prescription to be approximately 100 nm in size or even the size within 1000 nm processed by nanotechnology [77]. Nano-TCM may be an important direction towards TCM's modernization and internationalization. The activity of some Chinese medicines processed into nanodrugs has been greatly improved, which could significantly increase their bioavailability and targeting, decrease the using time, and largely reduce the toxic side effects [78–80]. Acupuncture combined with nano-TCM could offer a great potential in some type of characteristic acupuncture therapies improvement. As the development of nano-TCM, it would offer more options for acupoint injection thus expanding the range of the clinical applications. Acupoint external application is a specific therapeutic method that drugs, especially traditional Chinese herbs, are made into plaster to be applied on certain acupoints. The mechanism of this therapy relies on percutaneous absorption. Compared with traditional Chinese medicine, nano-TCM exhibits stronger penetrating ability due to small particle size and large selection of adsorption capacity, which could penetrate the skin barrier. After a certain modification of nanodrug carriers, it could promote sustained release of active constituent of the TCM, thus prolonging the curative effect [81].

Acupoint catgut embedding therapy is referred to infix several surgical chromic catgut sutures into the acupoints or subcutaneous tissue with a specialized needle under aseptic precautions. It is widely used in obesity, perimenopausal syndrome, chronic urticaria, depressive neurosis, and refractory insomnia [82]. Currently, the commonly used threads in clinical practice could be divided into medical catgut thread, absorbable surgical suture, and medicated suture [83]. In most cases, this therapy is considered safe, but some individual could be allergic to catgut. Inspired by the application of nanomaterial in medicine, a nanosilver thread is applied to acupoint catgut embedding therapy. The nanosilver with the antibacterial effect is better than the conventional catgut in lower inflammatory response [84].

## 5. Conclusion and Perspectives

Fluorescent magnetic nanoparticles allow the application of magnetic properties together with the ability of fluorescence to visible, for example, PVS. Although there are still some differences between PVS and acupuncture medians, nanoparticles give a new resolution to explore the basis of acupuncture medians. Nanochips or nanosensors are promising for detecting and monitoring the changing of acupuncture induced bioactive molecules and the intensity and amount of stimulation. Under the circumstances, it will help to fill the gap in knowledge about the underlying molecular mechanisms of acupuncture treatment and gain the quantitative, objective acupuncture manipulation simulation data. Accordingly, the standard of acupuncture manipulation could be set up and conducive to attracting more and more people to adopt acupuncture therapy.

The meridians and acupoints are dynamic processes of receiving stimuli and regulating functional activities of the body. The molecular imaging is intended to visually display bioactive molecule level in physiology or monitoring the pathological dynamic process of the bioactive molecule level* in vivo *or* in situ*. Molecular imaging based on nanotechnology, especially the multimodal imaging, could be a powerful approach that provides more reliable and accurate detection of dynamic changing of meridians and acupoints.

By virtue of nanotechnology, the acupuncture needles could be innovated as multifunction toolbox. It could realize the idea of not only controlled drug delivery but also warming and invigorating the acupoint in the original basis. Microneedles, which were first conceptualized for controlled drug delivery, emphasize drug and vaccine delivery to the skin and deliver molecules into cells and nuclei. Acupuncture needles could be considered as a method for controlled drug delivery using microneedle for reference. The acupuncture needles are fabricated with nanoscale conical pores upon the surface of needles in which drugs could be filled, or just coated the drug on the surface of the needles. In that case, it can not only have the therapeutic effects of acupuncture stimulation but also bring the drug to the lesions directly.
